# Lack of Utility of Nasopharyngeal Swabs for Diagnosis of *Burkholderia pseudomallei* Pneumonia in Paediatric Patients

**DOI:** 10.1093/tropej/fmv109

**Published:** 2016-02-13

**Authors:** Pasco Hearn, Claudia Turner, Kuong Suy, Sona Soeng, Nicholas P. J. Day, Paul Turner

**Affiliations:** ^1^Cambodia-Oxford Medical Research Unit, Angkor Hospital for Child, Siem Reap, Cambodia; ^2^Nuffield Department of Clinical Medicine, University of Oxford, Oxford, UK; ^3^Angkor Hospital for Children, Siem Reap, Cambodia; ^4^Faculty of Tropical Medicine, Mahidol University, Bangkok, Thailand

**Keywords:** *Burkholderia pseudomallei*, melioidosis, paediatric, nasopharyngeal swab

## Abstract

Diagnosis of *Burkholderia pseudomallei* pneumonia in children is challenging. We investigated the utility of nasopharyngeal swabs taken from 194 paediatric patients on admission with radiologically proven pneumonia. Melioidosis was proven in 0.5% of samples tested and only in a third of those known to be bacteraemic with *B. pseudomallei.* It appears unlikely that culture of nasopharyngeal secretions is helpful to confirm *B. pseudomallei* pneumonia in paediatric patients.

## INTRODUCTION

*Burkholderia pseudomallei* is a Gram-negative saprophyte found in the wet soils of many tropical countries [1]. Infection occurs after environmental exposure, by inhalation, inoculation or ingestion and leads to melioidosis, an important cause of morbidity and mortality in Southeast Asia. The infection is typified by abscess formation and can lead to a variety of clinical presentations in children, ranging from suppurative parotitis to fulminant sepsis. Overall, the lungs are the most common site of infection with *B. pseudomallei* [2]. Pneumonia accounted for 29% of presentations in a recent review of 24 years of paediatric melioidosis from Northern Australia [3] and 8% of the first 39 cases identified at Angkor Hospital for Children (AHC), Cambodia [4]. The diagnosis can be confirmed with culture of blood, pus or sputum [5, 6]. Unfortunately, in the paediatric population, obtaining sputum for culture is often not possible. In its place, nasopharyngeal (NP) swabs have been used as diagnostic specimens in pneumonia cases. This approach has proven successful for detection of viral pathogens, for example respiratory syncytial virus, and bacterial species that do not normally colonize the nasopharynx, for example *Mycobacterium tuberculosis.* It has also been demonstrated previously that there appears to be no asymptomatic upper respiratory carrier state for *B. pseudomallei* [7], although symptomatic melioidosis of the nasopharynx has been described in a child from Singapore [8]. While throat swabs have been shown to be diagnostically useful in suspected melioidosis cases [9], no data exist for the utility of NP swabs. We sought to determine whether NP swabs would be useful for identifying *B. pseudomallei* infection in Cambodian children admitted to the hospital with radiologically proven pneumonia.

## MATERIALS AND METHODS

AHC is a paediatric hospital located in Siem Reap, a province in Northern Cambodia with a population of around 1 million. Patients admitted to AHC between 1 August 2013 and 31 July 2014, suspected of having meningitis, pneumonia or sepsis and who gave informed consent were enrolled into a prospective study of pneumococcal colonization and disease [10]. All enrolled patients had NP swabs taken as soon as possible after admission. The flocked nylon tip of each swab was excised immediately into a cryovial containing 1 ml skim milk-tryptone-glucose-glycerol (STGG) medium and transferred by cool box to a −80°C freezer, where it remained until thawed for this study. Patients had a blood culture taken at the discretion of the treating clinician and a lumbar puncture performed if signs of meningism were present. Those with suspected pneumonia had a chest X-ray taken, which was primarily read and interpreted by the AHC radiologist. These radiographs were later re-read by two study clinicians and interpreted according to the World Health Organization paediatric radiologic pneumonia criteria [11]. NP swab specimens from patients with radiologically confirmed pneumonia were re-examined in this study.

## LABORATORY METHODS

The limit of detection of *B. pseudomallei* in STGG before and after freezing at −80°C was determined using Miles and Misra methodology [12]. Serial dilutions of a clinical *B. pseudomallei* isolate were made in STGG: 10 µl of each dilution was inoculated in triplicate onto Ashdown’s agar and incubated at 37°C in air for 48 h to determine colony counts. A further 10 µl of each dilution was stored at −80°C for 48 h and afterwards inoculated into *B. pseudomallei* selective broth and then Ashdown’s agar to confirm viability after freezing.

NP swab-STGG samples were fully thawed, and 10 µl was cultured in selective broth for 48 h, before being streaked out onto Ashdown’s agar. Inoculated plates were incubated in air at 35–37°C for up to 96 h, with the presence or absence of growth recorded. *Burkholderia pseudomallei* was identified by colony morphology, Gram stain, *B. pseudomallei*-specific latex agglutination and biochemical testing.

## RESULTS

The limit of detection of *B. pseudomallei* in STGG was 83.3 CFU/ml and did not change following storage at −80°C.

Of the 1009 hospitalized study patients, 194 patients had radiologically proven pneumonia and are described further. They had a median age of 1.20 years (inter quartile range: 0.41–2.39, range: 0.08–14.43). Blood cultures were collected in 171 cases, and *B. pseudomallei* was isolated from three (1.8%) of these. *Burkholderia pseudomallei* was isolated in only 1 of the 194 (0.5%) NP swab samples tested. This positive sample was taken from a patient who was also found to be bacteraemic with *B. pseudomallei.* NP swabs from two other patients with *B. pseudomallei* bacteraemia were negative.

## DISCUSSION

During a year in a paediatric setting where melioidosis is endemic, routine use of NP swabs would have provided the diagnosis of melioidosis in 1 of 194 cases of radiologically proven pneumonia. In addition, of those found to be bacteraemic with *B. pseudomallei*, only a third would have been diagnosed by NP swab, which is in line with the overall sensitivity of 36% previously reported for throat swabs [9]. One explanation may be that *B. pseudomallei* was present in the nasopharynx at levels lower than the limit of detection. Alternatively, environmental exposure to the organism by inhalation may result in lung infection directly, rather than via transient NP colonization, as is the case for *Streptococcus pneumoniae.* The major limitation of this study was the small number of culture-proven melioidosis cases: to confirm generalizability, it will be important to validate our findings in a patient population with a higher proportion of bacteraemic pneumonia cases.

Currently, with this work from Cambodia, we conclude that culture of NP secretions is unhelpful for confirmation of *B. pseudomallei* pneumonia in paediatric patients.

## FUNDING

This study was funded by a grant from the Li Ka Shing University of Oxford Global Health Programme and by the Wellcome Trust as part of the Wellcome Trust - Mahidol University Oxford Tropical Medicine Research Programme.
